# Research progress in molecular pathology markers in medulloblastoma

**DOI:** 10.37349/etat.2023.00126

**Published:** 2023-02-28

**Authors:** Zixuan Zhou, Bingxin Zhu, Qingming Meng, Tong Zhang, Yihao Wu, Rutong Yu, Shangfeng Gao

**Affiliations:** 1 Department of Neurosurgery, Institute of Nervous System Diseases, The Affiliated Hospital of Xuzhou Medical University, Xuzhou 221002, Jiangsu, China; 2 Department of Neurosurgery, Xuzhou Children’s Hospital, Xuzhou Medical University, Xuzhou 221002, Jiangsu, China; Tianjin Medical University General Hospital, China

**Keywords:** Medulloblastoma, molecular pathology, children, diagnosis, targeted therapy

## Abstract

Medulloblastoma (MB) is the commonest primary malignant brain cancer. The current treatment of MB is usually surgical resection combined with radiotherapy or chemotherapy. Although great progress has been made in the clinical management of MB, tumor metastasis and recurrence are still the main cause of death. Therefore, definitive and timely diagnosis is of great importance for improving therapeutic effects on MB. In 2016, the World Health Organization (WHO) divided MB into four subtypes: wingless-type mouse mammary tumor virus integration site (WNT), sonic hedgehog (SHH), non-WNT/non-SHH group 3, and group 4. Each subtype of MB has a unique profile in copy number variation, DNA alteration, gene transcription, or post-transcriptional/translational modification, all of which are associated with different biological manifestations, clinical features, and prognosis. This article reviewed the research progress of different molecular pathology markers in MB and summarized some targeted drugs against these molecular markers, hoping to stimulate the clinical application of these molecular markers in the classification, diagnosis, and treatment of MB.

## Introduction

Medulloblastoma (MB) is a kind of embryonal neuroepithelial tumor and the commonest primary malignant tumor of the central nervous system (CNS) in children, accounting for approximately 20% of children’s brain tumors [1]. It mostly occurs in the cerebellum or dorsal brainstem [2, 3]. MB is more common in males, with a male-to-female ratio of about 1.5:1 [4]. The World Health Organization (WHO) classified five histological subtypes of MB in 2007: classic, desmoplastic/nodular, extensive nodularity, large cell, and anaplastic MB [5]. In 2016, WHO released the CNS tumor classification, dividing MB into four main molecular subtypes: wingless-type mouse mammary tumor virus integration site (WNT), sonic hedgehog [SHH; tumor protein P53 (*TP53*) mutant and *TP53* wild-type], group 3, and group 4 [6, 7]. In the new version of WHO classification of the CNS tumors, MBs not belonging to the WNT or SHH group are designated as “non-WNT/non-SHH”, which is a combination of the established molecular subgroups “non-WNT/non-SHH group 3” and “non-WNT/non-SHH group 4” [8]. This large category accounts for the majority of MB cases.

Each of the four subtypes has a unique genetic and epigenetic profile, which is associated with different demography and clinical features (Table 1) [2, 9–12]. Schwalbe et al. [12] divided MB into seven subclassifications, of which the WNT MB remained unchanged. The SHH MB was split into age-dependent subgroups, infant (< 4.3 years) and child (≥ 4.3 years). The group 3 MB and the group 4 MB were respectively split into high-risk and low-risk subgroups. Cavalli et al. [11] divided MB into twelve subclassifications. The WNT MBs were split into α (ubiquitous monosomy 6) and β (chromosome 6 intact) subgroups. The SHH MBs were categorized into SHH α, SHH β, SHH γ, and SHH δ subtypes based on DNA methylation profiling. SHH β and SHH γ affect predominantly infants, whereas SHH α and SHH δ mostly affect children and adults, respectively. SHH α and SHH β have greater metastatic potential in comparison with SHH γ and SHH δ subtypes. The group 3 MB and the group 4 MB were respectively split into α, β, and γ subtypes. The group 3 γ MB has a poorer prognosis than the other two subgroups, while the other three subtypes of group 4 MB have similar prognostic profiles and different genetic alterations.

**Table 1.** Demography, clinical and molecular characteristics of MB

**Subgroup**		**WNT**		**SHH**				**Non-WNT/non-SHH group 3**			**Non-WNT/non-SHH group 4**		

**Subtype**		**WNT**	**WNT**	**SHH**	**SHH**	**SHH**	**SHH**	**Group**	**Group**	**Group**	**Group**	**Group**	**Group**
		**α**	**β**	**α**	**β**	**γ**	**δ**	**3α**	**3β**	**3γ**	**4α**	**4β**	**4γ**
Demography and clinical features	Age at diagnosis (years, median)	10	20	8	1.9	1.33	26	4.82	7.55	5	8.22	10	7
	Subtype proportion (%)	70	30	29.1	15.7	21.1	34.1	46.5	25.7	27.8	30.1	33.4	36.5
	Metastases (%)	8.6	21.4	20	33	8.9	9.4	43.4	20	39.4	40	40.7	38.7
	Survival rate (5 years, %)	97	100	69.8	67.3	88	88.5	66.2	55.8	41.9	66.8	75.4	82.5
Molecular features	Gene mutation	*CTNNB1*, *TP53*, *DDX3X*, *MLL2/3*		*PTCH1*, *SMO*, *SUFU*, *TP53*, *DDX3X*, *CREBBP*, *MLL2/3*, *TERT*, *KDM6A*				*TERT*, *KDM6A*			*TERT*, *KDM6A*		
	Gene modification	*TNRC6C* methy		*TNRC6C* methy, *MXI1* methy, *IL8* methy				*TNRC6C* methy, *MXI1* methy, *IL8* methy			*TNRC6C* methy, *MXI1* methy, *IL8* methy, *Lmx1A* enhancer activation, *PRDM6* induction		
	Copy number variation	*OTX2* amp, *CDK6* amp		*MYCN* amp, *CDK6* amp, *PTEN* loss, *GLI2* amp				*MYC* amp, *OTX2* amp, *CDK6* amp, *KDM6A* loss, *KBTBD4* insertion			*MYCN* amp, *OTX2* amp, *CDK6* amp, *PTEN* loss, *KDM6A* loss, *KBTBD4* insertion		
	miRNA profile	miR-183, miR-206		miR-206				miR-592, miR-182, miR-193a, miR-183, miR-206			miR-592, miR-182, miR-183, miR-206		
	Other events	-		-				*GFI1*/*GFI1B* activation, *MYC* acetylation, and phosphorylation			*GFI1*/*GFI1B* activation, *ERBB4-SRC* activation		
Potential targeted drugs		WNT/β-catenin inhibitor		SMO inhibitor, GLI inhibitor, PI3K inhibitor, CDK4/6 inhibitor				CDK4/6 inhibitor, MYC inhibitor			CDK4/6 inhibitor		

amp: amplification; *CDK6*: cyclin-dependent kinases 6; *CREBBP*: cAMP-response element binding protein (CREB)-binding protein; *CTNNB1*: cadherin-associated protein beta 1; *DDX3X*: DEAD-box helicase 3 X-linked; *ERBB4-SRC*: Erb-b2 receptor tyrosine kinase 4 (ERBB4)-proto-oncogene tyrosine-protein kinase SRC (SRC); *GFI1*: growth factor independent 1; *GLI2*: glioma-associated oncogene homolog 2; *IL8*: interleukin 8; *KBTBD4*: Kelch repeat and broad-complex, tramtrack, and bric-a-brac domain containing 4; *KDM6A*: lysine-specific demethylase 6A; *Lmx1A*: LIM homeobox transcription factor 1, alpha; methy: methylation; miRNA: microRNA; *MLL2*/*3*: mixed-lineage leukemia 2/3; *MXI1*: max interactor 1; *MYC*: myelocytomatosis oncogene; *MYCN*: neuroblastoma derived MYC; *OTX2*: orthodenticle homeobox 2; *PI3K*: phosphatidylinositol 3-kinase; *PRDM6*: PR/SET domain 6; *PTCH1*: patched 1; *PTEN*: phosphatase and tensin homolog; *SMO*: smoothened; *SUFU*: suppressor of fused; *TERT*: telomerase reverse transcriptase; *TNRC6C*: trinucleotide repeat containing 6C; -: blank cell

In the past decade, a number of molecular markers have been identified in MB, and they have shown potential application value in pathological diagnosis, targeted therapy, or prognostic evaluation. This paper reviewed the research progress of molecular pathologic markers in different subtypes of MB and expected to provide a basis for the routine application of these molecular markers in the clinical management of MB.

## Genetic alterations of molecular pathology markers in MB

### Point mutation

#### CTNNB1

*CTNNB1* gene, encoding β-catenin, was located in the region of chromosome 3p21–22. It regulates cell proliferation and differentiation by binding to various proteins and plays a key role in embryonic development and tumorigenesis [13, 14]. Zurawel et al. [15] first discovered point mutations in the *CTNNB1* gene in MB, which was later confirmed to be mainly present in the WNT subtype [6, 16, 17]. *CTNNB1* exon 3 has four phosphorylation sites. Phosphorylated β-catenin is degraded through the ubiquitin-proteasome pathway, whereas mutations of these sites cause β-catenin accumulation in the cytoplasm, eventually migrating to the nucleus. There it binds and activates the T-cell factor (TCF)/lymphoid enhancer factor (LEF), leading to the upregulation of particular target genes [18, 19]. Combining β-catenin immunohistochemistry and *CTNNB1* exon 3 sequencing is a feasible, economical, and effective approach to identifying the WNT subtype of MB, and patients of this subtype have a relatively good prognosis [19].

#### PTCH1, SMO, and SUFU

Activation mutations in the SHH pathway can be found in almost all SHH MBs. The most frequently mutated genes are *PTCH1*, *SMO*, and *SUFU*, and their expressions are mutually exclusive in MB [20–22]. *PTCH1* is mainly expressed in mesenchymal cells and is involved in embryonic structure formation and tumorigenesis. SMO proteins are important signal converters in the SHH pathway, and their activity is negatively regulated by *PTCH1*. *SUFU* is a major inhibitory factor in the SHH pathway. *PTCH1* mutation is the most common mutation in the SHH MB, occurring in all age groups [3, 16], although *SMO* mutation almost always occurs in adults [23]. A subset of pediatric patients with SHH MBs (aged 3 years to 16 years) showed *GLI2* and *MYCN* amplification to be mutually exclusive with *PTCH*, but 30% of which harbored phylogenetic (Li-Fraumeni syndrome) or *TP53* mutations [24]. Germline *SUFU* mutation has recently been identified as a genetic background that could cause MB in infants under 3 years old; no *SUFU* mutation has been found in the adult SHH subtype [23, 25–27]. SHH subtype patients with *SUFU* germline mutation have a worse prognosis than other SHH MB patients [28].

#### TP53

Human *TP53* gene, located on the short arm of chromosome 17, is a tumor-suppressor gene that encodes the P53 protein. It is involved in a number of important biological processes, e.g., cell cycle progression, DNA repair, cell differentiation, and apoptosis [29]. The presence of diffuse, strong P53 immunoreactivity in MB usually indicates a potential *TP53* mutation [30]. In a cohort study of 108 cases of MB, mutations in the *TP53* gene were reported as an independent predictor of poor prognosis [31]. Zhukova et al. [32]. showed that the prognostic value of somatic *TP53* mutation was subtype-dependent in a larger cohort that included 553 cases of MB. Specifically, patients with WNT-subtype tumors who carry somatic *TP53* mutation have a good prognosis, whereas patients with SHH subtype tumors who carry the same mutation have a worse prognosis [3, 29, 31]. Moreover, the SHH MB could be further divided into *TP53* wild-type and *TP53* mutant; *TP53* status is the most significant risk factor in SHH MB [13, 29], especially in SHH α subtype [11]. The 5-year overall survival rates of SHH subtype patients with or without *TP53* mutation are 41% and 81%, respectively [29]. Therefore, the new WHO classification of the CNS tumors divides SHH subtype MB into *TP53* wild-type and *TP53* mutant subgroups.

#### DDX3X

Human genome encodes two functional *DDX3* genes: *DDX3X* and its homologous gene *DDX3Y* [33]. *DDX3X* gene is located on the X chromosome and could regulate different steps of RNA metabolism, such as RNA splicing, transcription, and translation initiation. In addition, *DDX3X* is involved in stress response, cell apoptosis, cell cycle progression, and viral infection [33–35]. The role of *DDX3X* in tumorigenesis and progression is quite complex, and it plays a dual role in multiple tumors [36]. Downregulation of *DDX3X* promotes stem cell-like properties and tumorigenesis in hepatocellular carcinoma cells [37], while the upregulation of *DDX3* was observed in distant breast cancer metastases and correlated with poor prognosis [38, 39]. *DDX3X* functions as a tumor-suppressor gene in MB [13, 40]; its functional deletion mutation increased the incidence and severity of tumor formation in mouse models of WNT and SHH MBs [41]. *DDX3X* mutation is common in adult SHH MB patients, but it is reported to be very rare in the pediatric SHH subtype [23, 25, 26]. *DDX3Y* gene is located on the Y chromosome and plays a significant role in male fertility [33]. There have currently been no studies connecting *DDX3Y* with the development of MB.

#### CREBBP

*CREBBP* gene is involved in the transcriptional co-activation of many transcription factors, and its expression product is a nuclear protein that binds to the CREB. *CREBBP* plays a key role in embryonic development, cell growth control, and homeostasis maintenance through chromatin remodeling and transcription factor recognition [42]. *CREBBP* has been found to be almost completely mutated in adult SHH subtype MB [40]. Similar to the *DDX3X* mutation, *CREBBP* mutation is rare in pediatric SHH patients [11, 26, 43]. The loss of *CREBBP* acts synergistically with SHH signals to enhance SHH pathway output and drive tumor growth in MB [42]. This provides a new direction for targeted therapy of SHH MB.

#### MLL2 and MLL3

*MLL2* and *MLL3* are genes encoding histone-lysine *N*-methyltransferases involved in the methylation of histone 3 lysine 4 (H3K4) [44]. Histone methyltransferases affect heterochromatin formation, gene imprinting, and transcriptional regulation. *MLL2* and *MLL3* are tumor-suppressor genes that are inactivated by mutation [45]. In an early study, their mutations were mainly found in WNT or SHH subtype MB [44, 45], but later, Robinson et al. [40] reported a low incidence of *MLL2* mutations in group 3 MB. The discrepancy may be caused by a small sample size and a lack of subtype-specific analysis. Therefore, further studies using larger numbers of MB cases will be needed to reveal the specific relationship between the dysfunction of *MLL2*/*3* signaling and the sub-classification and prognosis of MB.

#### TERT-promoter mutation

Telomerase is an RNA-dependent DNA polymerase that can prolong the telomere DNA to maintain telomere homeostasis. Maintaining telomere length is a key step for cancer cells to overcome telomere shortening and induce cell senescence [46]. TERT is the rate-limiting catalytic subunit of telomerase. *TERT*-promoter mutation leads to the upregulation of *TERT* transcription, which enables cancer cells to avoid cell senescence and increase their replication potential. *TERT*-promoter mutation is the most common recurrent somatic point mutation in MB [46], occurring mainly in adult patients with SHH and WNT MB. It is interesting that *TERT*-promoter mutation in the SHH subtype was associated with a higher overall survival rate and lower incidence of tumor metastasis [47], while group 4 MB patients with *TERT*-promoter mutation had lower overall survival rates than those with *TERT*-promoter wild-type [48]. In WNT and group 3 subtypes of MB, *TERT*-promoter mutations appear to have no effect on overall survival [46]. Though the molecular basis for these differences in survival is unclear, the status of the *TERT*-promoter may provide a new biomarker for subtype classification and targeted therapy in MB.

### Copy number variation

#### MYC/MYCN amplification

*MYC* and *MYCN* induce cell proliferation and malignant transformation together with other oncogenes or tumor suppressors [49], and they are the two most frequently amplified oncogenes in MB. *MYC* and *MYCN* amplifications account for 5–10% of sporadic MB and have a high incidence in large cell subtypes [50]. *MYC* amplification is a hallmark alteration almost exclusively found in group 3 MB [51] and predicts an extremely poor prognosis [52, 53]. *MYCN* amplification is enriched in SHH and group 4 MB [54]. Combined ectopic expression of *MYCN* and SHH promotes the formation of cerebellar MB in mice after birth [54, 55]. *MYCN* amplification is associated with poor prognosis in SHH MB [23, 52, 55, 56]. However, neither *MYCN* gain nor amplification was associated with poor survival in group 4 MB [57].

#### OTX2 amplification

*OTX2* gene is composed of 5 exons, of which the first two are non-coding, and the last three encode *OTX2*. *OTX2* was previously identified as a potential oncogene for some malignancies, but recently it has been identified as a driver gene in MB [58]. Due to gene amplification [59], *OTX2* is highly expressed in WNT and non-WNT/non-SHH MB, although it is low or absent in the SHH subtype [60, 61]. The downregulation of OTX2 expression can inhibit the growth of MB cells *in vitro* [62]. Overexpression of OTX2 directly drives MB cell proliferation by targeting cell-cycle genes [63, 64]. Screening for OTX2 overexpression is becoming an integral part of establishing a molecular classification scheme in MB [62], although the correlation between the expression of OTX2 and patient prognosis has not been investigated yet.

#### CDK6 amplification

CDK6, a serine/threonine kinase, has strong effects on cell cycle progression [65]. CDK6 activation promotes cell cycle progression through the phosphorylation of substrates, including retinoblastoma protein (pRb) and transcription factors with roles in proliferation and differentiation [66]. Recently, some studies have shown that amplification of *CDK6* gene is a vital feature of group 4 MB [13, 48]. A genome-wide analysis of DNA copy number in 47 cases of MB showed that *CDK6* amplification was significantly associated with poor prognosis in MB [67]. Therefore, *CDK6* amplification/overexpression may be used as a biological marker for molecular stratification and therapeutic interventions in MB patients.

#### PTEN loss

*PTEN* is the main inhibitor of the PI3K signaling pathway, and PI3K activation is the main driver of most human cancers [68]. Frequent allele loss of *PTEN* in MB results in low expression of *PTEN*, which was associated with a low survival rate in a transgenic mouse model of MB [69]. Homozygous deletions of *PTEN* have been described in SHH MB [51]. Low expression of PTEN could identify high-risk patients with adverse outcomes in the SHH subtype, but not in the remaining MB subgroups [70]. In contrast, PTEN is highly expressed in group 4 MB and could be used to differentiate group 3 and group 4 subtypes [70], but the underlying mechanism has not yet been elucidated.

#### KDM6A mutation and loss

*KDM6A* [also known as ubiquitously transcribed tetratricopeptide repeat on chromosome X (*UTX*)], a tumor-suppressor gene encoding histone 3 lysine 27 (H3K27) demethylase, plays a vital part in determining cell fate and cell differentiation during development [13, 40 , 44, 71]. Robinson et al. [40] first reported the high-frequence mutation of *KDM6A* in MB. *KDM6A* mutations are enriched in group 4 MB and are identified with lower frequencies in SHH and group 3 MB [13, 23, 72]. In addition, *KDM6A* copy-number loss was often found in female non-WNT/non-SHH MB patients [40]. Although the exact mechanism is not clear, *KDM6A* gene mutation promotes tumorigenesis in a mouse model of MB [73], providing novel insights into the function of *KDM6A* in MB.

#### KBTBD4 gene insertion

*KBTBD4* gene encodes a Kelch protein belonging to a family of ubiquitin-ligase adapters that facilitates the ubiquitination of target substrates. *KBTBD4* gene insertions are located at a hotspot region (codons 308–313) and have been reported exclusively in non-WNT/non-SHH MBs [25, 48, 49]. However, researchers from Brazil analyzed a series of 111 MBs, including 48 cases from the non-WNT/non-SHH subtype; none of the 48 harbored any *KBTBD4* mutations at the hotspot region [74]. This may have been the result of population differences or small sample size. Therefore, future studies are warranted to assess the frequency and role of *KBTBD4* mutations in MB.

#### GLI2 amplification

*GLI* is the end effector of hedgehog (HH) signaling and promotes transcription of HH-target genes, which could regulate cell survival, invasion, and angiogenesis, as well as stem cell self-renewal and epithelial-mesenchymal transition [75–79]. It has been found that *GLI2* amplification exists in SHH MB [23, 25] and frequently co-occurs with *TP53* loss (defined as SHH α subtype), predicting a worse prognosis in patients with this subtype of MB [80]. Additionally, GLI2 is positively regulated by the PI3K/AKT pathway [81], which is also mutated in a subset of SHH MB patients [23].

#### Chromosome abnormalities

Chromosome abnormalities are often observed in MBs, particularly those classified as group 3 and group 4 subtypes [9]. Isochromosome (iso) 17q is the commonest cytogenetic change in group 4 MB, although it is also seen in group 3 MB [54, 60]. Shih et al. [57] reported that iso 17q was a statistically significant predictor of poor outcomes in group 3 but not in group 4 MB. In addition, chromosome 17 gain and chromosome 11 loss were found to be good prognostic factors in group 4 MB [56, 82]. These findings indicate that chromosome 17 aberration is a subtype-specific molecular biomarker in MB.

### Gene modification

#### TNRC6C methylation

TNRC6 proteins, including TNRC6A, TNRC6B, and TNRC6C, serve as scaffolding proteins within miRNA-induced silencing complex and therefore play an important role in miRNA-mediated gene silencing [83]. Whole-genome methylation sequencing of circulating tumor DNA (ctDNA) in cerebrospinal fluid showed that DNA methylation in the 3’-untranslated region (UTR) of *TNRC6C* was significantly increased in all subgroups of MB, and can be used as a potential prognostic marker to predict clinical outcomes in patients with this tumor [84].

#### MXI1 and IL8 methylation

MXI1 is a negative regulator of the MYC family of proteins [85], and IL8 has potential involvement in chemokine signaling and angiogenic processes in tumor development [86]. Recently the methylation of *MXI1* and *IL8* was identified as a novel independent high-risk biomarker in the survival models of SHH and non-WNT/non-SHH MB patients [87]. Incorporation of DNA methylation events into current risk-stratification schemes significantly improved the accuracy of survival prediction, which has important implications for future risk-adapted clinical disease management in MB.

#### Lmx1A enhancer activation

Lmx1A is a LIM homeobox transcription factor 1, alpha previously shown to function as a critical regulator of cell-fate determination in cerebellar development [88]. Lin et al. [89] reported that both *Lmx1A* enhancer activity and expression are important for the identification of group 4 MB. Their chromatin immunoprecipitation (ChIP)-sequencing data supported Lmx1A as a master regulator of the transcription factor in the transcriptional program of group 4 MB.

#### PRDM6 induction by enhancer hijacking

PRDM6 belongs to the PRDM family of transcriptional repressors, a family that is essential for the growth of smooth muscle cells [90]. It was reported that *PRDM6* gene expression was markedly upregulated in a subset of group 4 MB patients, due to “enhancer hijacking” induced DNA rearrangement [25]. Further studies are needed to confirm the function of *PRDM6* as an oncogene in this subtype of MB.

### Post-transcriptional/translational modification

#### GFI1/GFI1B transcriptional activation

*GFI1B* is a paralog of *GFI1*. Both genes functioned as sustained natural apophyseal glides (SNAG) domain-containing zinc finger transcriptional repressors essential for a variety of developmental processes [91]. *GFI1* and *GFI1B* were identified as prominent oncogenes specifically activated in non-WNT/non-SHH MB, and somatic genomic rearrangements together with mutually exclusive activation of *GFI1* and *GFI1B* were found in approximately one-third of group 3 MB patients [92]. These oncogenes are now considered the commonest enhancer hijacker in this subtype of MB [13, 25, 92], and they may have a synergistic effect on *MYC* gene amplification in promoting the malignant progression of MB [92]. Therefore, *GFI1* and *GFI1B* are promising biomarkers for molecular typing and targeted therapy in non-WNT/non-SHH MB.

#### Aberrant ERBB4-SRC signaling

*ERBB4* is the only member of the ERBB receptor family with growth-inhibiting properties. According to the Cancer Cell Line Encyclopedia database, its messenger RNA (mRNA) expression is only present in a small fraction of tumor cell lines, whereas the other ERBB receptors are highly expressed in the majority of tumor cell lines [93]. The controversies around the anti- or pro-oncogenic role of *ERBB4* can in part be explained by the multiple ligands that can activate *ERBB4*, its numerous intracellular phosphorylation sites, the presence of alternative splice variants, the different intracellular signaling pathways affected, and the different downstream responses in different cell types and different disease stages. Using quantitative (phospho)-proteomics in primary human MBs, Forget et al. [94] unraveled distinct post-transcriptional regulation leading to highly divergent oncogenic signaling and kinase activity profiles, e.g., aberrant ERBB4-SRC [a key protein tyrosine kinase to regulate receptor tyrosine kinase (RTK) signaling] signaling in group 4 MB. These findings indicated that *ERBB4* promoted MB malignance and could serve as a therapeutic target in group 4 subtype.

#### MYC post-translational modification

*MYC* amplification is a “hallmark” of *MYC*-active MB, but not all tumors of this type have *MYC* amplification [25, 95]. Archer et al. [96] quantitatively profiled global proteomes and phospho-proteomes in 45 MB samples, and found that increased post-translational modifications of MYC, e.g., acetylation and phosphorylation, are associated with poor outcomes in group 3 MB, and correlate with the increased phosphorylation of protein kinase, DNA-activated, catalytic subunit (PRKDC). Inhibiting the activity of PRKDC sensitizes *MYC*-active MB cells to radiation [96], which offers a new strategy for the treatment of group 3 MB.

#### miRNA

miRNAs are a class of endogenous non-coding small RNAs with a length of 18–22 basepair (bp) that regulate the expression of target genes by inducing mRNA degradation or translation inhibition [97]. They control basic cellular processes such as development, differentiation, metabolism, proliferation, and apoptosis. Non-transcriptional expression of miRNAs is associated with the development and progression of a variety of cancers, and such expression changes can be caused by mutations, methylation, deletions, and gains in the miRNA coding region [98]. miR-124 was first reported to be positively associated with the survival of MB patients, and it can inhibit tumor cell growth by targeting CDK6 [99, 100]. Kunder et al. [101] carried out a miRNA expression analysis in different subtypes of MB and identified miR-592 and miR-182 as surrogate markers for non-WNT/non-SHH group 3/group 4 MB. The two mRNAs were also useful for risk stratification of this category of MB. They later found that restoration of miR-193a expression or overexpression of miR-206 suppressed tumor cell growth in MB cells [102, 103]. In addition, high expression of miR-182 and miR-183 was positively associated with the metastasis of non-SHH MB [104]. These studies suggest that miRNA profiling might be a promising marker for risk stratification, molecular typing, and prognosis estimation of MB [105].

## Molecular targeted therapy in MB

Surgical resection, combined with radiotherapy and chemotherapy, is still the main mode for the treatment of MB, but the efficacy is limited. Thanks to the advances in molecular pathological markers of MB, molecular targeted therapy is becoming a promising strategy to overcome this type of pediatric brain tumor. Here, we mainly documented the targeted therapy studies related to the molecule markers mentioned above.

### SMO inhibitor

Thirty percent of MBs show hyperactivation of SHH signaling pathways [16, 106]. Vismodegib (GDC-0449, 879085-55-9, Genentech, US), the first United States Food and Drug Administration (US FDA)-approved SMO antagonist, has shown therapeutic efficacy in recurrent SHH subtype adult patients, although this subtype is also prone to drug resistance [107]. As demonstrated in phase I and II clinical trials [43], another SMO inhibitor, sonidegib (LDE-225, 956697-53-3, Novantis, Switzerland), seems to be more effective than vismodegib in treating SHH driven adult and pediatric MB. However, both drugs are ineffective on tumors driven by mutations in SHH pathway genes downstream of SMO, implying that infants (SHH β and γ) and children (SHH α) with SHH driven MB are unlikely to benefit from these drugs. In summary, SMO inhibitors act only on tumors with mutations in genes upstream of the SMO pathway. Adult patients with SHH MB are the best candidates for this therapy compared with children and infants [43].

### GLI inhibitor

GLI transcription factors are critical mediators of the HH pathway, which is usually activated in SHH MB. Glioma-associated oncogene antagonist-61 (GANT61), the first GLI antagonist, has been proven to inhibit cell migration, invasion, and proliferation while enhancing cell apoptosis in human MB cells [108]. Arsenic trioxide (ATO) can interact with GLI1 to inhibit GLI1 transcriptional activity [109] and could promote GLI2 degradation in MB cells [110]. The effectiveness of ATO as a HH pathway inhibitor has been tested in several preclinical tumor models [109, 111, 112]. ATO, used alone or in combination with other anticancer drugs, may represent a valuable therapeutic option to treat SHH MB, particularly those harboring drug-resistant *SMO* mutations [109, 110, 113].

### PI3K inhibitor

Alterations of the signaling pathway of the intracellular lipid kinase PI3K are known to play a crucial role in MB by regulating cellular growth, proliferation, and cell survival [114]. It has been suggested that the PI3K signaling pathway can positively regulate the expression of GLI2, an end effector of HH signaling [75–77]. Targeting both PI3K and HH pathways is considered a promising therapeutic strategy for SHH MB. Treatment of MB cells with the HH inhibitor vismodegib and the PI3K inhibitor BEZ235 (915019-65-7, LC laboratories, US) significantly suppressed cell growth and survival, and increased cisplatin-mediated cytotoxicity [115]. Two clinical trials are recruiting patients for the treatment of recurrent MB using the PI3K inhibitor samotolisib (NCT03213678, NCT03155620, 1386874-06-1, Selleckchem, US).

### WNT/β-catenin inhibitor

PRI-724, a *CREBBP*-*CTNNB1* interacting antagonist, is currently being involved in phase I clinical trials of pancreatic cancer and hepatitis-C-virus-infected cirrhosis (ClinicalTrials.gov identifier NCT01764477 and NCT02195440) [116], and is expected to be applied in the treatment of WNT subtype MB. WNT subtype of MB usually has a good prognosis because patients’ blood-brain barrier is leaky, which allows better delivery of chemotherapy drugs to tumor cells [117]. Inhibiting WNT signaling would improve the integrity of the blood-brain barrier, making tumors more resistant to chemotherapy. Therefore, drugging to the WNT/β-catenin pathway requires much caution.

### CDK4/6 inhibitor

The CDK4/6-meidated signaling pathway has been recently identified as a druggable target for all non-WNT MBs [118]. As an inhibitor specifically targeting CDK4/6, palbociclib (571190-30-2, Pfizer, US) has been demonstrated as an effective therapeutic drug for MB, especially the group 3 subtype with *MYC* amplification [118]. It is currently being used in clinical trials for all subtypes of MB and other childhood brain cancers (NCT02255461) [119]. In addition, a number of clinical trials are recruiting MB patients to explore the efficacy of CDK4/6 inhibitors in combination with conventional chemotherapy, such as abemaciclib (1231929-97-7, Eli Lilly and Company, US) and temozolomide (NCT04238819, 85622-93-1, Merck Sharp & Dohme, US), ribociclib (1256963-02-6, Novantis, Switzerland) and gemcitabine (NCT03434262, 95058-81-4, Eli Lilly and Company, US). Interestingly, Daggubati et al. [120] reported that decreased ribosomal protein expression caused the resistance of SHH MB to CDK6 inhibition, which provides a rationale for the combination therapy to treat this subtype of MB.

### MYC inhibitor

*MYC* signature activation is found to be associated with poor outcomes in group 3 MB [52, 53], while there has not been a clear path for targeted therapy. Notably, a novel bromodomain inhibitor JQ1 (1268524-70-4, Selleckchem, US) was developed to interrupt the hyper-transcriptional activity of *MYC*-driven MB cells and xenografts [121, 122]. As yet, there are no bromodomain inhibitors with FDA approval for use in MB.

### Other potential drug targets

Targeted drugs against other molecular markers, such as *DDX3X*, *KDM6A*, and *MLL2*/*3*, have not yet emerged and require further research. Small-molecule drugs targeting various miRNAs are also a new option for the treatment of MB [105, 123], but they are still in the pre-clinical stages.

## Conclusions

With the development of more efficient and accurate molecular biology technologies, molecular pathology markers have shown an increasingly important role in MB. As summarized in Table 2, these molecular markers are helpful in the pathology diagnosis, risk stratification, or prognostic evaluation in MB. Some of them have been extensively studied and become the classification criteria for MB. For example, β-catenin immunohistochemistry is routinely used to identify the WNT MB; TP53 mutation is a high-risk factor for SHH MB. *MYC* amplification is specific for group 3 MB and predicts poor prognoses, and *CDK6* amplification is a vital feature of group 4 MB. Other markers are needed to be more comprehensively studied to clarify their specific roles in MB, such as MLL2/3, TERT, KDM6A, KBTBD4, and ERBB4. It should also be noted that some molecular markers are interrelated or mutually exclusive. In pediatric MB, *TP53* mutation often occurs simultaneously with *GLI2* and *MYCN* amplification. *SUFU* mutation is mutually exclusive with *PTCH1* and *SMO* mutation, and it mainly occurs in infant MB, while *DDX3X*, *CREBBP*, and *TERT*-promoter mutation is frequently found in adult MB.

**Table 2.** Summary of molecular pathology markers in MB

**Gene**	**Status**	**MB subtypes**	**Prognosis**	**References**
*CTNNB1*	Mutation	WNT	Good	[19]
*PTCH1*/*SMO*	Mutation	SHH	Dependent on TP53	[3, 16, 23]
*SUFU*	Germline mutation	SHH	Poor	[28]
*TP53*	Mutation	WNT	Good	[3, 21, 29]
		SHH	poor	[3, 13, 21, 29]
*DDX3X*	Mutation	WNT, SHH	Unknown	[13, 40, 41]
*CREBBP*	Mutation	SHH	Unknown	[40, 42]
*MLL2*	Mutation	WNT, SHH	Unknown	[44, 45]
*MLL3*	Mutation	WNT, SHH	Unknown	[44, 45]
*TERT*	Promoter mutation	WNT	Unaffected	[46]
		Group 3		
		SHH	Good	[47]
		Group 4	Poor	[48]
*MYC*	Amplification	Group 3	Poor	[51–53]
	Post-translational modifications	Group 3	Poor	[96]
*MYCN*	Amplification	SHH	Poor	[23, 52, 55, 56]
		Group 4	Unknown	[57]
*OTX2*	Amplification	WNT, non-WNT/non-SHH	Unknown	[62–64]
*CDK6*	Amplification	All groups	Poor	[13, 48, 67]
*PTEN*	Loss	SHH	poor	[51, 70]
		Group 4	Unknown	[70]
*KDM6A*	Copy number loss	non-WNT/non-SHH	Unknown	[40]
	Mutation	SHH, non-WNT/non-SHH		[13, 23, 72]
*KBTBD4*	Insertion	non-WNT/non-SHH	Unknown	[25, 48, 49, 74]
*GLI2*	Amplification	SHH	Poor	[23, 25, 80]
*Chromosome 11*	Loss	Group 4	Good	[56, 82]
*Chromosome 17*	iso 17q	Group 3	Poor	[54, 57, 60]
	Gain	Group 4	Good	[56, 82]
*TNRC6C*	DNA methy	All groups	Poor	[84]
*MXI1*	DNA methy	Non-WNT	Poor	[87]
*IL8*	DNA methy	Non-WNT	Poor	[87]
*Lmx1A*	Enhancer activation	Group 4	Poor	[89]
*PRDM6*	Enhancer hijacking	Group 4	Unknown	[25]
*GFI1*	Transcriptional activation	non-WNT/non-SHH	Unknown	[13, 25, 92]
*GFI1B*	Transcriptional activation	non-WNT/non-SHH	Unknown	[13, 25, 92]
*ERBB4*	Post-transcriptional regulation	Group 4	Poor	[94]

methy: methylation

The advances in molecular pathology markers provide deep insights into the tumorigenesis mechanism and targeted therapy of MB. The molecular mechanism driving WNT and SHH MB has been deeply studied, and some drugs targeting SHH pathways (e.g., vismodegib) and PI3K pathways (e.g., BEZ235) are being tested in clinical trials. Other drugs targeting the WNT/SHH MB, e.g., WNT/β-catenin inhibitor and GLI inhibitor, have also been investigated extensively, but they are still in the preclinical stage. The non-WNT/non-SHH MB, accounting for more than two-thirds of all MBs, has the highest metastasis rate. The underlying mechanism of this category of MB is largely unknown, which limits the development of targeted drugs. MYC activation is found to be associated with poor outcomes in group 3 MB, while there has not been a clear path for targeted therapy. The CDK4/6 inhibitor, palbociclib, is the sole drug that has entered the clinical trial for the treatment of non-WNT/non-SHH MB, especially group 3 subtype. Chromosome abnormalities are often found in group 3 and group 4 MB, such as iso 17q, chromosome 17 gain, and chromosome 11 loss. The alteration in chromosomes can be easily detected by sequencing and provide useful information for the prognosis of non-WNT/non-SHH MB, but the drug targets are so far lacking due to the complexity and heterogeneity of this type of MB. Therefore, further investigations are needed to identify homogeneous subtypes within the non-WNT/non-SHH MB, which may simplify the development of targeted drugs.

## Abbreviations

ATO: arsenic trioxide
CDK6: cyclin-dependent kinases 6
CNS: central nervous system
CREBBP: cAMP-response element binding protein-binding protein
CTNNB1: cadherin-associated protein beta 1
DDX3X: DEAD-box helicase 3 X-linked
ERBB4-SRC: Erb-b2 receptor tyrosine kinase 4-proto-oncogene tyrosine-protein kinase SRC
GFI1: growth factor independent 1
GLI2: glioma-associated oncogene homolog 2
HH: hedgehog
IL8: interleukin 8
iso: isochromosome
KBTBD4: Kelch repeat and broad-complex tramtrack, and bric-a-brac domain containing 4
KDM6A: lysine-specific demethylase 6A
Lmx1A: LIM homeobox transcription factor 1, alpha
MB: medulloblastoma
miRNA: microRNA
MLL2/3: mixed-lineage leukemia 2/3
MXI1: max interactor 1
MYC: myelocytomatosis oncogene
MYCN: neuroblastoma derived myelocytomatosis oncogene
OTX2: orthodenticle homeobox 2
PI3K: phosphatidylinositol 3-kinase
PRDM6: PR/SET domain 6
PTCH1: patched 1
PTEN: phosphatase and tensin homolog
SHH: sonic hedgehog
SMO: smoothened
SUFU: suppressor of fused
TERT: telomerase reverse transcriptase
TNRC6C: trinucleotide repeat containing 6C
TP53: tumor protein P53
WHO: World Health Organization
WNT: wingless-type mouse mammary tumor virus integration site

## Declarations

### Copyright

© The Author(s) 2023.
